# Subclinical Changes in Cardiac Functional Parameters as Determined by Cardiovascular Magnetic Resonance (CMR) Imaging in Sleep Apnea and Snoring: Findings from UK Biobank

**DOI:** 10.3390/medicina57060555

**Published:** 2021-05-31

**Authors:** Adrian Curta, Holger Hetterich, Regina Schinner, Aaron M. Lee, Wieland Sommer, Nay Aung, Mihir M. Sanghvi, Kenneth Fung, Elena Lukaschuk, Jackie A. Cooper, José Miguel Paiva, Valentina Carapella, Stefan Neubauer, Stefan K. Piechnik, Steffen E. Petersen

**Affiliations:** 1 Clinic of Radiology, Ludwig-Maximilian University Hospital, 81377 Munich, Germany; hetterich@rad-ro.de (H.H.); regina.schinner@med.uni-muenchen.de (R.S.); w.sommer@smart-reporting.com (W.S.); 2 NIHR Biomedical Research Centre at Barts, William Harvey Research Institute, Queen Mary University of London, London E1 4NS, UK; a.lee@qmul.ac.uk (A.M.L.); n.aung@qmul.ac.uk (N.A.); m.sanghvi@qmul.ac.uk (M.M.S.); kenneth.fung@bartshealth.nhs.uk (K.F.); jackie.cooper@qmul.ac.uk (J.A.C.); j.paiva@qmul.ac.uk (J.M.P.); s.e.petersen@qmul.ac.uk (S.E.P.); 3 Cardiovascular Medicine, John Radcliffe Hospital, University of Oxford, Oxford OX3 9DU, UK; elena.lukaschuk@cardiov.ox.ac.uk (E.L.); carapella@gmail.com (V.C.); stefan.neubauer@cardiov.ox.ac.uk (S.N.); stefan.piechnik@cardiov.ox.ac.uk (S.K.P.)

**Keywords:** cardiac MRI, sleep apnea, heart, epidemiology

## Abstract

*Background and Objectives:* Obstructive sleep apnea (OSA) is a common disorder with an increased risk for left ventricular and right ventricular dysfunction. Most studies to date have examined populations with manifest cardiovascular disease using echocardiography to analyze ventricular dysfunction with little or no reference to ventricular volumes or myocardial mass. Our aim was to explore these parameters with cardiac MRI. We hypothesized that there would be stepwise increase in left ventricular mass and right ventricular volumes from the unaffected, to the snoring and the OSA group. *Materials and Methods:* We analyzed cardiac MRI data from 4978 UK Biobank participants free from cardiovascular disease. Participants were allocated into three cohorts: with OSA, with self-reported snoring and without OSA or snoring (*n* = 118, 1886 and 2477). We analyzed cardiac parameters from balanced cine-SSFP sequences and indexed them to body surface area. *Results:* Patients with OSA were mostly males (47.3% vs. 79.7%; *p* < 0.001) with higher body mass index (25.7 ± 4.0 vs. 31.3 ± 5.3 kg/m²; *p* < 0.001) and higher blood pressure (135 ± 18 vs. 140 ± 17 mmHg; *p* = 0.012) compared to individuals without OSA or snoring. Regression analysis showed a significant effect for OSA in left ventricular end-diastolic index (LVEDVI) (β = −4.9 ± 2.4 mL/m²; *p* = 0.040) and right ventricular end-diastolic index (RVEDVI) (β = −6.2 ± 2.6 mL/m²; *p* = 0.016) in females and for right ventricular ejection fraction (RVEF) (β = 1.7 ± 0.8%; *p* = 0.031) in males. A significant effect was discovered in snoring females for left ventricular mass index (LVMI) (β = 3.5 ± 0.9 g/m²; *p* < 0.001) and in males for left ventricular ejection fraction (LVEF) (β = 1.0 ± 0.3%; *p* = 0.001) and RVEF (β = 1.2 ± 0.3%; *p* < 0.001). *Conclusion:* Our study suggests that OSA is highly underdiagnosed and that it is an evolving process with gender specific progression. Females with OSA show significantly lower ventricular volumes while males with snoring show increased ejection fractions which may be an early sign of hypertrophy. Separate prospective studies are needed to further explore the direction of causality.

## 1. Introduction

Obstructive sleep apnea (OSA) is a common disorder with an estimated prevalence of 10–17% in males and 3–9% in females aged between 30 and 70 [1]. A recent literature based analysis calculated that almost a billion individuals worldwide could be affected by OSA [2]. Snoring is a common symptom in patients with OSA [3] and a strong, independent predictor of the disease [4]. In these individuals, narrow upper airways caused by obesity, anatomical tightness and nocturnal loss of muscle tone leads to repeated upper-airway occlusions with resultant hypoxemia and hypercapnia. This triggers a chemoreflex-induced sympathetic response which persists into daytime and leads to an increased risk for cardiovascular disease [5], particularly hypertension and congestive heart failure [3,6,7]. A recent study showed an association between OSA and myocardial hypertrophy in females [7]. Furthermore, OSA is associated with left atrial dilation [8], arrhythmia [9] and stroke [10].

Pulmonary hypertension has also been associated with OSA although it has not been as thoroughly investigated as left ventricular (LV) alterations [11]. By increasing the pressure in the right ventricle (RV), pulmonary hypertension leads to dilation and hypertrophy of the RV. Although pulmonary hypertension is common in OSA with a reported prevalence of 20–44% [12], RV heart failure seems to be rare [13]. However, data are limited to echocardiography studies with well-known limitations regarding the assessment of RV function. Cardiovascular magnetic resonance (CMR) imaging is the standard of reference for the assessment of right and left ventricular function [14]. So far this technique has not been used to systematically assess the impact of OSA and snoring on cardiac function.

This study examines whether there is an alteration in LV and RV structure and function, as determined by CMR, in subjects with snoring or OSA in a population-based cohort free from known cardiovascular disease.

## 2. Materials and Methods

### 2.1. Study Sample

For this study, we analyzed the data provided by the UK Biobank, a population-based prospective cohort study. From 2006 to 2010, approximately 500,000 people aged between 40 and 69 years were recruited from across the United Kingdom to participate in this study. The study was approved by the local ethics committee (Ref: 16/NW/0274) and was conducted in accordance with the declaration of Helsinki. Participants provided detailed medical data through interviews and questionnaires and underwent measurements of multiple clinical parameters and provided bio samples for further detailed analysis. A subgroup of 100,000 participants were to undergo a comprehensive imaging protocol including an MRI scan of the heart. The study protocol used was described in detail previously [15].

**Figure 1:**
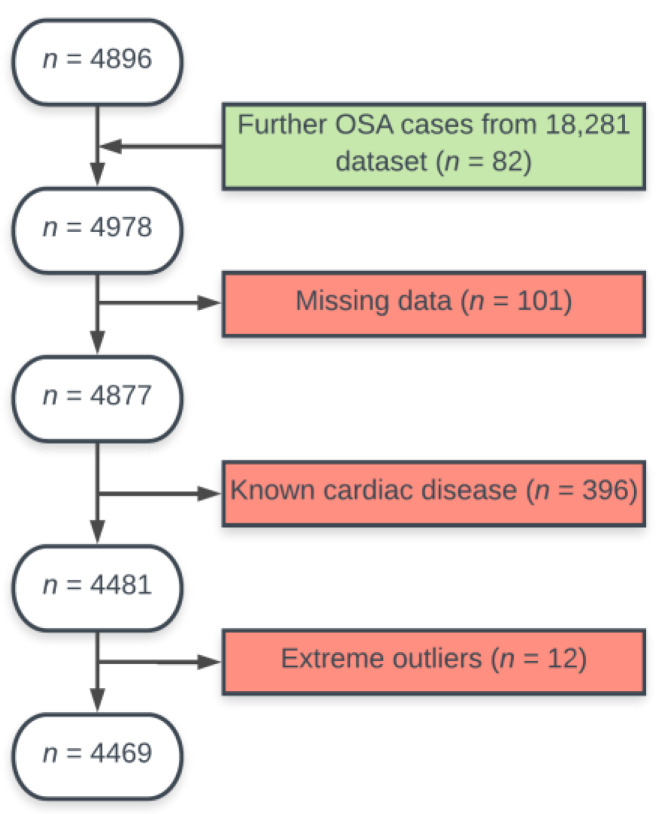
Case selection flow chart.

Two core laboratories in London and Oxford, UK, conducted the manual image analysis of a total of 4,877 CMR examinations [16]. As the number of individuals with OSA in this dataset was unexpectedly low (*n* = 36), we conducted a search in our complete, non-evaluated dataset of 18,281 individuals. From this data we could detect a further 82 individuals with OSA who met inclusion criteria. We excluded participants with missing CMR data (*n* = 6) and individuals with known cardiac or cardiovascular disease (*n* = 396). A total of 13 individuals with known OSA and 230 individuals with snoring did not meet the inclusion criteria. Extreme outliers with values of over three times the interquartile range (*n* = 12) were also excluded. The exclusion process is displayed in Figure 1. The remaining 4481 individuals were evaluated for further analysis.

### 2.2. Assessment of OSA and Snoring

Information on OSA status was derived from two sources, the National Health Service—Hospital Episode Statistics (NHS-HES) data and standardized interviews as part of the UK Biobank assessment. In the case of NHS-HES data, an electronic file was created for each patient admitted to an NHS institution either as in- or outpatient where clinical diagnoses are then subsequently recorded.

### 2.3. CMR Imaging and Evaluation

CMR was performed on a clinical 1.5 Tesla Magnetom Aera tomograph, (Siemens Healthcare, Erlangen, Germany). In order to determine left and right ventricular functional parameters, balanced cine steady-state-free-precession sequences were acquired in the 2-, 3-, 4-chamber view and the short axis. The evaluation of the CMR sequences was performed using the cvi^42^ software suite (Circle Cardiovascular Imaging Inc., Calgary, AB, Canada). Detailed information on the full protocol has been described elsewhere [16]. Body surface area (BSA), adjusted LV end-diastolic index (LVEDVI), LV end-systolic volume index (LVESVI), LV mass index (LVMI), RV end-diastolic volume index (RVEDVI) and RV end-systolic volume index (RVESVI) were measured. LV ejection fraction (LVEF) and RV ejection fraction (RVEF) as well as BSA-adjusted LV stroke volume index (LVSVI) and RV stroke volume (RVSVI), were then calculated.

### 2.4. Covariates

The recruited individuals were questioned for underlying disease and health risk factors and underwent a physical examination. Blood pressure and heart rate were measured twice at the same appointment at a time interval of slightly over a minute. Both measurements were performed in a seated position in the left upper arm whenever possible. Diabetes status was derived from an electronic questionnaire. Individuals who answered, “Do not know” were asked again in an interview by trained staff. Smoking status and daytime dozing were acquired via electronic questionnaire. Height and weight were measured prior to imaging.

### 2.5. Statistical Analysis

Differences between OSA status groups were compared using Kruskal–Wallis or Mann–Whitney-U tests. For categorical parameters results are presented as frequency counts and percentages and differences between OSA status groups were compared using a chi-square test. We used multiple linear regression to compensate for known confounders: OSA status groups, age, systolic and diastolic blood pressure, diabetes mellitus, body mass index (BMI) and smoking status. Smoking was used as a binary variable, dividing participants into a smoking and a non-smoking group. The confidence interval, where applicable, was set at 95%. As this is an explorative analysis, all parameters were compared to an explorative significance level of alpha = 5% without adjusting for multiple comparisons; a *p*-value of <0.05 was regarded as statistically significant. The analysis was performed by an independent statistician using SAS Version 9 (SAS Institute Inc., Cary, NC, USA).

## 3. Results

### 3.1. Study Population

The evaluated 4,481 individuals were divided into three groups: (a) known OSA (*n* = 118; 2.6%), (b) self-reported snoring but no known OSA (*n* = 1886; 42.1%) and (c) neither known OSA nor snoring (*n* = 2477; 55.3%). A total of 53.4% of the participants were female, self-reported origin was European in 94.2% of cases, mean age was 55.2 ± 7.6 years. There were significantly more male participants in the OSA and snoring groups compared to the unaffected individuals (79.7%; 56.8% and 37.3%, respectively; *p* < 0.001). There was no significant age difference between the groups (55.3 ± 7.3 years vs. 55.1 ± 7.5 years vs. 55.2 ± 7.7 years; *p* = 0.840). BMI was significantly higher in the OSA and the snoring group compared to the unaffected group (31.3 ± 5.3 kg/m² vs. 27.6 ± 4.3 kg/m² vs. 25.7 ± 4.0 kg/m²; *p* < 0.001). Daytime dozing was significantly higher in the OSA and the snoring group compared to the unaffected group (*p* < 0.001). Both systolic and diastolic blood pressure showed a highly significant difference when comparing the snoring group to the unaffected group (*p* < 0.001) and a slightly significant difference when comparing the OSA group to the control (*p* = 0.012 and *p* = 0.045, respectively). There were significant differences in diabetes status in the OSA and snoring group compared to the control (*p* < 0.001 and *p* = 0.005). Detailed demographic parameters are provided in Table 1.

### 3.2. Left and Right Ventricular Parameters

**Figure 2:**
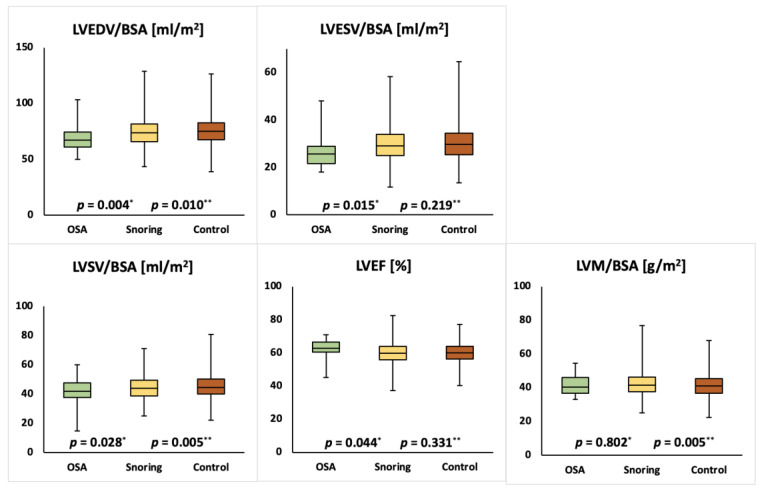
Comparison of left ventricular ejection fraction (LVEF) and end-diastolic volume index (LVEDVI), end-systolic volume index (LVESVI), stroke volume index (LVSVI) and mass index (LVMI) in female individuals with obstructive sleep apnea (OSA), snoring or none of the above. *p*-values for OSA vs. unaffected (*) and snoring vs. unaffected (**) are reported.

**Figure 3:**
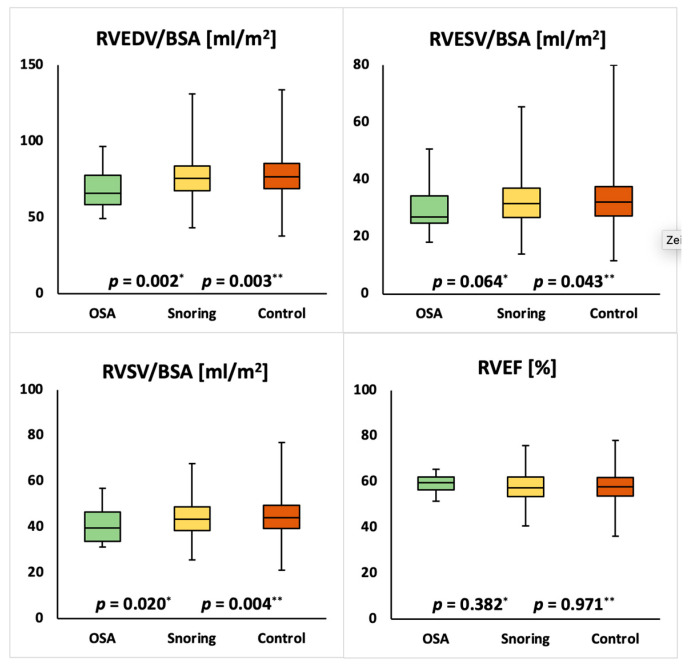
Comparison of right ventricular ejection fraction (RVEF) and end-diastolic volume index (RVEDVI), end-systolic volume index (RVESVI) and stroke volume index (RVSVI) in female individuals with obstructive sleep apnea (OSA), snoring or none of the above. *p*-values for OSA vs. unaffected (*) and snoring vs. unaffected (**) are reported.

**Figure 4:**
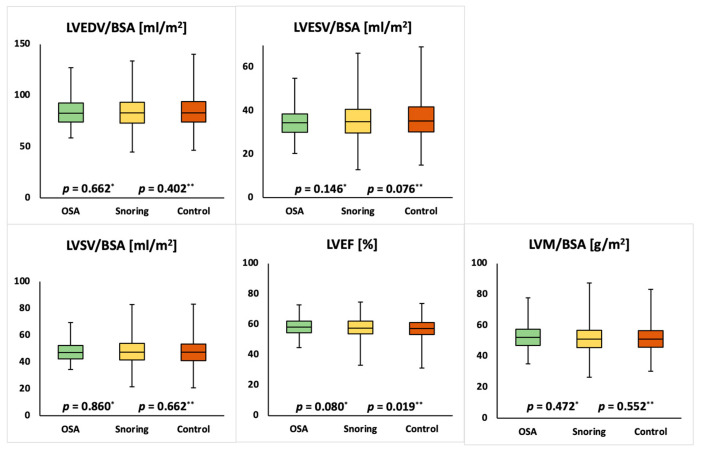
Comparison of left ventricular ejection fraction (LVEF) and end-diastolic volume index (LVEDVI), end-systolic volume index (LVESVI), stroke volume index (LVSVI) and mass index (LVMI) in male individuals with obstructive sleep apnea (OSA), snoring or none of the above. *p*-values for OSA vs. unaffected (*) and snoring vs. unaffected (**) are reported. No significant difference is shown between individuals with OSA and not affected individuals.

**Figure 5:**
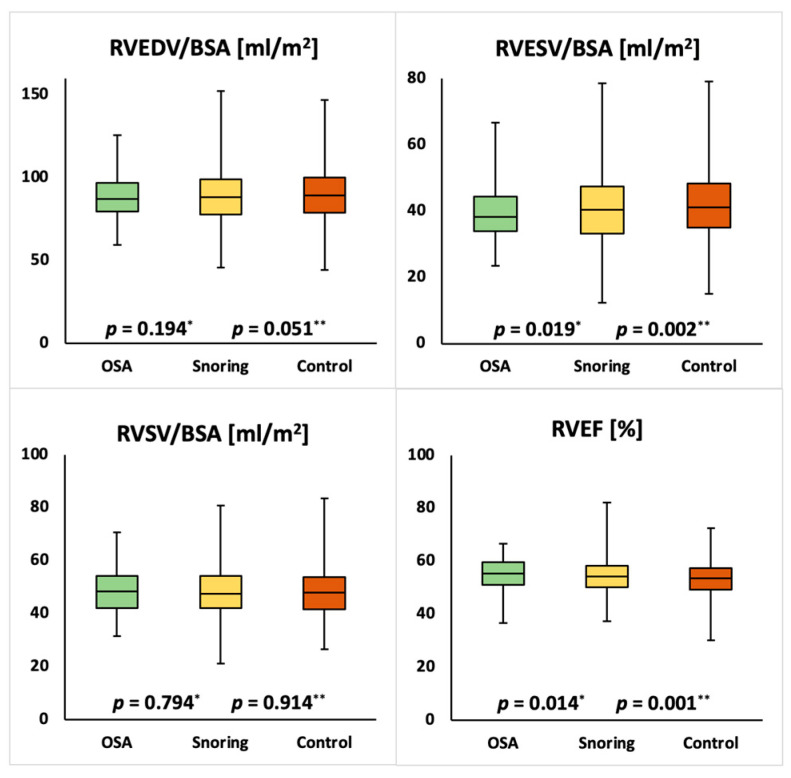
Comparison of right ventricular ejection fraction (RVEF) and end-diastolic volume index (RVEDVI), end-systolic volume index (RVESVI) and stroke volume index (RVSVI) in male individuals with obstructive sleep apnea (OSA), snoring or none of the above. *p*-values for OSA vs. unaffected (*) and snoring vs. unaffected (**) are reported.

Mean cardiac parameters for the whole study population were within normal ranges [17]. Compared to the unaffected group females with snoring showed significantly lower LVEDVI (75.6 ± 11.7 mL/m² vs. 74.3 ± 11.8 mL/m²; *p* = 0.010), LVSVI (45.4 ± 7.6 mL/m² vs. 44.4 ± 7.7 mL/m²; *p* = 0.005), RVEDVI (77.8 ± 12.6 mL/m² vs. 76.1 ± 12.3 mL/m²; *p* = 0.003), RVESVI (32.9 ± 8.1 mL/m² vs. 32.2 ± 7.8 mL/m²; *p* = 0.043) and RVSVI (44.8 ± 7.5 mL/m² vs. 43.9 ± 7.3 mL/m²; *p* = 0.004) as well as a higher LVMI (41.4 ± 6.8 g/m^2^ vs. 42.2 ± 6.8 g/m^2^; *p* = 0.005). Compared to the unaffected group females with OSA showed significantly lower LVEDVI (75.6 ± 11.7 mL/m² vs. 68.7 ± 11.9 mL/m²; *p* = 0.004), LVESVI (30.2 ± 6.9 mL/m² vs. 26.7 ± 7.3 mL/m²; *p* = 0.015), LVSVI (45.4 ± 7.6 mL/m² vs. 41.9 ± 9.9 mL/m²; *p* = 0.028) and RVEDVI (77.8 ± 12.6 mL/m² vs. 69.5 ± 13.1 mL/m²; *p* = 0.002) and a significantly higher LVEF (60.2 ± 5.6% vs. 62.6 ± 5.1%; *p* = 0.044). Compared to the unaffected group males with snoring showed significantly lower RVESVI (42.3 ± 10.3 mL/m² vs. 40.7 ± 10.0 mL/m²; *p* = 0.002) and significantly higher LVEF (57.2 ± 6.2% vs. 57.8 ± 6.1%; *p* = 0.019) and RVEF (53.5 ± 6.1% vs. 55.1 ± 5.9%; *p* = 0.001). Compared to the unaffected group males with OSA showed significantly lower RVESVI (42.3 ± 10.3 mL/m² vs. 39.7 ± 8.5 mL/m²; *p* = 0.002) and significantly higher LVEF (57.2 ± 6.2% vs. 57.8 ± 6.1%; *p* = 0.019) and RVEF (53.5 ± 6.1% vs. 54.5 ± 6.1%; *p* = 0.014). The detailed results are displayed in Table 2 and Table 3 and Figure 2, Figure 3, Figure 4 and Figure 5.

### 3.3. Regression Analysis

Regression analysis, after adjusting for known confounders, showed a significant effect in LVEDVI (β = −4.9 ± 2.4 mL/m²; *p* = 0.040) and RVEDVI (β = −6.2 ± 2.6 mL/m²; *p* = 0.016) in females and for RVEF (β = 1.7 ± 0.8%; *p* = 0.031) in males when comparing the OSA group to the unaffected group.

A significant regression effect was shown when comparing the snoring group to unaffected participants in females for LVMI (β = 3.5 ± 0.9 g/m²; *p* < 0.001) and in males for LVEF (β = 1.0 ± 0.3%; *p* = 0.001) and RVEF (β = 1.2 ± 0.3%; *p* < 0.001).

## 4. Discussion

In this population-based study we analyzed cardiac parameters in individuals without known cardiac disease for their associations with OSA and snoring. After modifying for known confounders, regression analysis showed an association of the OSA status to lower LVEDVI and RVEDVI in females and to a higher RVEF in males. Furthermore, we observed an association of snoring with higher LVM for females and with higher LVEF and RVEF for males.

Analyzing an otherwise healthy population enables us to discern discreet subclinical effects on cardiac parameters which should represent alterations in the early stages of the disease. Nevertheless, the prevalence of known OSA in our study group is very low compared to current literature with 1.1% in males and 0.2% in females. The current estimated prevalence in the ages 30 to 70 is about 10–17% in men and 3–9% in women [1]. This suggests that there is a high number of individuals with unknown OSA in the snoring group as self-reported snoring was shown to be an independent predictor of OSA, especially in women with a sensitivity of up to 71% and a specificity of up to 89% [4].

The exclusion of individuals with known cardiac disease does not seem to play a role in the low prevalence of known OSA as the total prevalence including these individuals was 1.9% in male and 0.3% in female participants. Individuals with known cardiac disease show a high cardiac comorbidity with OSA, which has been described as a marker of heart failure severity [18,19]. Especially in individuals with acute myocardial infarction there has been a reported prevalence of OSA of up to 65.7% [20].

In concordance with current literature, the OSA and the snoring group were mostly comprised of overweight males [21,22]. There was a significantly higher incidence of diabetes, smoking and hypertension in the snoring group, which is in keeping with recent literature [7].

The significant decrease in LVEDVI and RVEDVI in female individuals with OSA could be attributed to an increase in myocardial stiffness. Farré et al. demonstrated in an animal study that intermittent hypoxia, as in OSA, leads to remodeling of the extracellular matrix with an increase in myocardial stiffness which could play a great role in the pathogenesis of OSA [23]. In this case the diastolic relaxation of the ventricles would be slightly impaired due to the increased stiffness.

Advanced stages of OSA have been associated with a decrease in LVEF under 50% [24]. In male individuals, we demonstrated a positive correlation of the snoring status to LVEF and RVEF in the snoring group and to RVEF in the OSA group with values still in the normal ranges. A slight increase in EF has been described in early, asymptomatic cases of LV hypertrophy [25]. LVMI was significantly increased in female individuals with snoring compared to the control.

The small, subclinical effect sizes for LVM were expected, as individuals with known heart disease had been excluded. Nevertheless, the measured values in the OSA and snoring group were in the upper two quartiles of normal ranges which could point to initial subclinical alterations.

In contrast to other studies, a significant increase in heart rate was not observed in the OSA or the snoring group [11]. We also could not verify the reported greater risk for OSA in ethnic groups other than Caucasian, for example, African Americans and Hispanics, as our population was mostly Caucasian [26,27].

There are several studies exploring LV and RV dysfunction using echocardiography. In most cases this is achieved by E- and A- wave analysis, in some cases also reporting LV and RV diameters [21,28,29]. A few studies analyzed other parameters such as LVSV [30] and RVEF [31]. A thorough literature research yielded only a few studies analyzing cardiac functional parameters with CMR in patients with OSA. Sharma et al. compared cardiac functional parameters of patients with OSA and patients with OSA and COPD without finding any significant difference in cardiac functional parameters between both groups [32].

Our study further explores data from cross-sectional studies describing LV hypertrophy and RV dilation [21,28,29,30,31]. Measurements were performed using CMR which is the current standard of reference for cardiac functional analysis providing accurate and reproducible data [14].

### 4.1. Limitations

The main limitation of our study was the lack of polysomnogram data by which diagnosis of OSA is usually established. This allows for an unknown number of undiagnosed individuals in the snoring group. In addition, the detection of snoring by interview poses a further limitation as individuals without someone to observe them snoring could unknowingly give a false answer. Transition from snoring to OSA is a process that is often not observed during early stages of the disease, resulting in a great number of individuals with undiagnosed OSA [26]. Polysomnogram data would also have enabled us to divide study participants into degrees of OSA and let us define a control group with excluded OSA.

Reevaluation of data after completion of manual segmentation of all 100,000 datasets should lead to an increase in the number of individuals with OSA and thus to a higher statistical power for a more precise evaluation of subclinical changes.

Dependence on questionnaires to obtain information on participant characteristics and chronic diseases is a further limitation to our study.

Even though we excluded individuals with cardiac disease there are other non-cardiac conditions that can influence CMR parameters that may not have been accounted for.

The cross-sectional study design does not allow conclusions regarding causality.

### 4.2. Strengths

The large number of participants in our study lets us demonstrate subtle differences in cardiac parameters in the snoring group.

The study with the next highest number of participants was conducted in the setting of the Multiethnic Study of Atherosclerosis and included ca. 1,400 participants. It showed an increase in LVM and a decrease in LVEF in OSA patients without evaluating ventricular volumes or RVEF [33]. As this study analyzed individuals who had undergone polysomnography, over 80% of the included individuals were diagnosed with OSA which is noticeably higher than the prevalence reported in current literature [22], pointing to a potential selection bias.

The exclusion of participants with known cardiovascular disease allows us to dismiss the most common comorbidity of OSA, and to adjust our results for known confounders. Our data was standardized throughout the study allowing for direct comparison between study participants without the need for data modification.

## 5. Conclusions

In this cross-sectional study, we compared cardiac parameters as determined by CMR in participants with OSA, snoring or none of the above. After adjusting for confounders, we demonstrated decreased LVEDVI and RVEDVI in females as probable initial stiffening of the myocardium. In males, OSA correlated to an increase in RVEF while snoring correlated to an increase in both LVEF and RVEF probably as a more premature sign of initial hypertrophy. The small effect sizes are of no clinical significance but may point to initial alterations in cardiac function due to OSA. The different results in both genders in this pilot study could point to a gender specific progression of the disease in the initial stages. Furthermore, our study suggests that OSA is greatly underdiagnosed and in need of better prevention strategies. This hypothesis remains to be tested once the full evaluation of CMR-datasets is completed and could be a subject for future prospective studies.

## Figures and Tables

**Table 1:** Study participant information; snoring vs. unaffected * and OSA vs. unaffected **.

Parameter	Unaffected (*n* = 2477)	Snoring (*n* = 1886)	*p*-Value *	OSA (*n* = 118)	*p*-Value **
Female gender	1153 (62.7%)	815 (43.2%)	<0.001	24 (20.3%)	<0.0001
Age (years)	55.2 ± 7.7	55.1 ± 7.5	0.458	55.3 ± 7.3	0.914
Height (cm)	169 ± 9	171 ± 10	<0.001	174 ± 9	<0.001
Weight (kg)	72.6 ± 13.9	80.2 ± 15.0	<0.001	93.6 ± 17.0	<0.001
BMI (kg/m²)	25.7 ± 4.0	27.6 ± 4.3	<0.001	31.3 ± 5.3	<0.001
Body surface area (m²)	1.8 ± 0.2	1.9 ± 0.2	<0.001	2.1 ± 0.2	<0.001
Systolic blood pressure (mmHg)	135 ± 18	138 ± 17	<0.001	140 ± 17	0.012
Diastolic blood pressure (mmHg)	78 ± 10	80 ± 10	<0.001	80 ± 10	0.045
Pulse rate (/s)	70 ± 11	70 ± 11	0.038	71 ± 12	0.081
**Smoking Status**					
Current	95 (3.8%)	90 (4.8%)	<0.001	7 (5.9%)	0.097
Never	1570 (63.4%)	1058 (56.1%)		64 (54.2%)	
Previous	783 (31.6%)	715 (37.9%)		46 (39.0%)	
Unknown	29 (1.2%)	23 (1.2%)		1 (0.8%)	
**Daytime dozing**					
Often	38 (1.5%)	55 (2.9%)	<0.001	6 (5.1%)	<0.001
Sometimes	423 (17.1%)	435 (23.1%)		39 (33.1%)	
Never/rarely	1990 (80.3%)	1377 (73.0%)		71 (60.2%)	
No answer	26 (1.0%)	19 (1.0%)		2 (1.7%)	
Diabetes mellitus	89 (3.6%)	101 (5.4%)	0.005	18 (15.3%)	<0.001

**Table 2:** Descriptive statistics of left and right ventricular cardiac parameters snoring vs. unaffected * and OSA vs. unaffected ** in female subjects. Significant results are marked in bold. Reported values are left ventricular mass index (LVMI), end-diastolic volume index (LVEDVI), end-systolic volume index (LVESVI), ejection fraction (LVEF) and stroke volume index (LVSVI) and right ventricular end-diastolic volume index (RVEDVI), end-systolic volume index (RVESVI), ejection fraction (RVEF) and stroke volume index (RVSVI).

Parameter	Unaffected	Snoring		OSA	
	Mean ± SD	Mean ± SD	*p*-Value *	Mean ± SD	*p*-Value **
LVMI (g/m^2^)	41.4 ± 6.8	42.2 ± 6.8	**0.005**	41.7 ± 6.1	0.802
LVEDVI (mL/m^2^)	75.6 ± 11.7	74.3 ± 11.8	**0.010**	68.7 ± 11.9	**0.004**
LVESVI (mL/m^2^)	30.2 ± 6.9	29.9 ± 6.9	0.219	26.7 ± 7.3	**0.015**
LVEF (%)	60.2 ± 5.6	59.9 ± 5.8	0.331	62.6 ± 5.1	**0.044**
LVSVI (mL/m^2^)	45.4 ± 7.6	44.4 ± 7.7	**0.005**	41.9 ± 9.9	**0.028**
RVEDVI (mL/m^2^)	77.8 ± 12.6	76.1 ± 12.3	**0.003**	69.5 ± 13.1	**0.002**
RVESVI (mL/m^2^)	32.9 ± 8.1	32.2 ± 7.8	**0.043**	29.9 ± 8.4	0.064
RVEF (%)	57.9 ± 6.0	57.9 ± 5.9	0.971	59.0 ± 4.2	0.382
RVSVI (mL/m^2^)	44.8 ± 7.5	43.9 ± 7.3	**0.004**	41.2 ± 7.0	0.020

**Table 3:** Descriptive statistics of left and right ventricular cardiac parameters snoring vs. unaffected * and OSA vs. unaffected ** in male participants. Significant results are marked in bold. Reported values are left ventricular mass index (LVMI), end-diastolic volume index (LVEDVI), end-systolic volume index (LVESVI), ejection fraction (LVEF) and stroke volume index (LVSVI) and right ventricular end-diastolic volume index (RVEDVI), end-systolic volume index (RVESVI), ejection fraction (RVEF) and stroke volume index (RVSVI).

Parameters	Unaffected	Snoring		OSA	
	Mean ± SD	Mean ± SD	*p*-Value *	Mean ± SD	*p*-Value **
LVMI (g/m^2^)	51.6 ± 8.5	51.8 ± 8.8	0.552	52.2 ± 8.2	0.472
LVEDVI (mL/m^2^)	84.4 ± 14.9	83.8 ± 14.6	0.402	83.7 ± 13.1	0.662
LVESVI (mL/m^2^)	36.2 ± 8.7	35.5 ± 8.6	0.076	34.8 ± 7.4	0.146
LVEF (%)	57.2 ± 6.2	57.8 ± 6.1	**0.019**	58.3 ± 5.3	0.080
LVSVI (mL/m^2^)	48.1 ± 9.3	48.3 ± 9.3	0.662	48.3 ± 8.0	0.860
RVEDVI (mL/m^2^)	90.5 ± 15.9	89.1 ± 16.0	0.051	88.3 ± 13.1	0.194
RVESVI (mL/m^2^)	42.3 ± 10.3	40.7 ± 10.0	**0.002**	39.7 ± 8.5	**0.019**
RVEF (%)	53.5 ± 6.1	54.5 ± 6.1	**0.001**	55.1 ± 5.9	**0.014**
RVSVI (mL/m^2^)	48.3 ± 9.1	48.4 ± 9.0	0.914	48.6 ± 8.3	0.794

## Data Availability

Published data can be reviewed on the UK Biobank data repository.
